# Knockout of butyrophilin subfamily 1 member A1 (*BTN1A1*) alters lipid droplet formation and phospholipid composition in bovine mammary epithelial cells

**DOI:** 10.1186/s40104-020-00479-6

**Published:** 2020-07-03

**Authors:** Liqiang Han, Menglu Zhang, Zhiyang Xing, Danielle N. Coleman, Yusheng Liang, Juan J. Loor, Guoyu Yang

**Affiliations:** 1 College of Animal Science and Veterinary Medicine, Henan Agricultural University, Zhengzhou, 450002 PR China; 2 Department of Animal Sciences and Division of Nutritional Sciences, University of Illinois, Urbana, Illinois 61801 USA

**Keywords:** Lipid droplet, Mammary epithelial cell, Milk fat globule, Phospholipid

## Abstract

**Background:**

Milk lipids originate from cytoplasmic lipid droplets (LD) that are synthesized and secreted from mammary epithelial cells by a unique membrane-envelopment process. Butyrophilin 1A1 (BTN1A1) is one of the membrane proteins that surrounds LD, but its role in bovine mammary lipid droplet synthesis and secretion is not well known.

**Methods:**

The objective was to knockout BTN1A1 in bovine mammary epithelial cells (BMEC) via the CRISPR/Cas9 system and evaluate LD formation, abundance of lipogenic enzymes, and content of cell membrane phospholipid (PL) species. Average LD diameter was determined via Oil Red O staining, and profiling of cell membrane phospholipid species via liquid chromatography-tandem mass spectrometry (LC-MS/MS).

**Results:**

Lentivirus-mediated infection of the Cas9/sgRNA expression vector into BMEC resulted in production of a homozygous clone *BTN1A1*^*(−/−)*^. The LD size and content decreased following *BTN1A1* gene knockout. The mRNA abundance of fatty acid synthase (*FASN*) and peroxisome proliferator-activated receptor-gamma (*PPARG*) was downregulated in the *BTN1A1*^(−/−)^ clone. Subcellular analyses indicated that BTN1A1 and LD were co-localized in the cytoplasm*. BTN1A1* gene knockout increased the percentage of phosphatidylethanolamine (PE) and decreased phosphatidylcholine (PC), which resulted in a lower PC/PE ratio.

**Conclusions:**

Results suggest that *BTN1A1* plays an important role in regulating LD synthesis via a mechanism involving membrane phospholipid composition.

## Introduction

The process of milk fat secretion begins in the endoplasmic reticulum of mammary epithelial cells (MEC) from which lipid droplets (LD) begin to form [1]. During secretion from cells into milk, LD are progressively enveloped by the apical plasma membrane forming milk fat globules (MFG) with sizes ranging from 0.2–15 μm [2–4]. Inside MFG, there is a lipid core surrounded by a complex phospholipid- and protein-coated membrane (MFGM) [5]. The size and features of MFG have received particular interest due to their influence on the manufacturing properties and nutritional quality of dairy products [6–8]. It has been suggested that intracellular LD formed by mammary epithelial cells are related to the size of secreted MFG [9, 10] and, thus, the mechanism regulating LD size in the cytoplasm is relevant to the properties of MFG.

The abundance of some proteins in the MFGM of MEC affects formation of LD [11–14]. Proteomic analysis of the composition of the MFGM revealed that the most-abundant proteins include butyrophilin (BTN1A1), perilipin 2 (PLIN2; formerly adipophilin) and xanthine dehydrogenase (XDH) [15]. The BTN1A1 protein accounts for more than 20% of total protein in the MFGM [3, 16]. Abundance of these proteins increases during lactation in the bovine mammary gland [17]. McManaman et al. [1] proposed that BTN may function as an integral receptor for cytoplasmic LD, and budding of the droplets at the cell surface initiates formation of complexes between BTN and XDH. Robenek et al. [18] suggested that a crucial feature of MFG secretion is the establishment of a network of adhesion sites containing BTN in the secretory granule monolayer. Clearly, BTN1A1 proteins are involved in MFG formation, but little is known about the mechanism whereby *BTN1A1* aids in LD formation in bovine MEC.

The clustered regularly interspaced short palindromic repeats (CRISPR) and CRISPR-associated 9 (Cas9) system can be used for genome editing in mammalian cells via single-guide RNA (sgRNA) [19–21]. Using sgRNA leads to Cas9-mediated cleavage of the target DNA and the protospacer adjacent motif, resulting in double-stranded breaks [22]. In mammalian cells, double-stranded breaks (DSB) are mostly repaired by the non-homologous end-joining pathway that enables efficient construction of knockout alleles by introducing small insertions or deletions that result in loss of target protein expression [23]. The aim of this study was to investigate the effect of knockout of *BTN1A1* on LD formation using the CRISPR/Cas9 system. The LD size was measured by Oil Red O staining, and profiling of cell membrane phospholipid species was analysed via liquid chromatography-tandem mass spectrometry.

## Materials and methods

### Bovine mammary epithelial cell isolation and culture

Bovine mammary epithelial cell (BMEC) were isolated and purified as described previously [24]. Briefly, mammary tissue (150 mg) was harvested from 4 to 5 years old late-lactation dairy cows and immediately transported to the laboratory. Samples were washed with DPBS (D8662, Sigma) and cut into three 1 mm pieces. Tissue was transferred with tweezers onto clean cell culture dishes at 37 °C in an atmosphere of 5% CO_2_ and 95% air. After 4 h, 5 mL of basal medium was added into the culture. The basal medium was replaced with fresh medium every 24 h until cells were thoroughly distributed across the bottom of the dish. Basal medium was composed of DMEM/F12 (12400–024, Gibco, New York, USA) and 10% (v/v) fetal bovine serum (16000–044, Gibco, New York, USA). Subsequently, BMEC were enriched by selective detachment with 0.25% trypsin (Gibco, Grand Island, NY, USA). After 3 min of enzyme digestion, detached fibroblasts were removed by washing with DPBS and epithelial cells attached to the dish were allowed to grow by addition of fresh medium. The BMEC were continuously purified using the same method. The purified BMEC (1 × 10^6^ cells/mL) were suspended in freezing medium for cryopreservation in liquid nitrogen.

After thawing, the BMEC were cultured in basal medium similar to that of Peterson et al. [25] with modifications. The basal medium was composed of DMEM/F12 with 10% fetal bovine serum, insulin (5 μg/mL, I6634, Sigma, St. Louis, MO, USA), hydrocortisone (1 μg/mL, H0888, Sigma), transferrin (5 μg/mL, T1428, Sigma), progesterone (1 μg/mL, P8783, Sigma), and epidermal growth factor (100 ng/mL, SRP3027, Sigma). At approximately 24 h before applying treatments (approximately 70–80% confluence), cells were cultured in lactogenic medium as reported by Kadegowda et al. [26] with modifications. The lactogenic medium was DMEM/F12 without serum, containing bovine Serum Albumin (1 mg/mL, A1933, Sigma), insulin (5 μg/mL, I6634, Sigma), hydrocortisone (1 μg/mL, H0888, Sigma) and prolactin (1 μg/mL, 682-PL, R&D Systems) to induce protein synthesis.

We designed primers and measured mRNA abundance of β-casein in BMEC by RT-PCR to confirm the lactogenic state of BMEC. In order to determine the suitable puromycin tolerance concentration in BMEC, cells were seeded in 35-mm cell culture dish and when they approached 70–80% confluence, puromycin (P8833, Sigma) was added to the culture medium at a final concentration of 0, 2.5, 5.0, 7.5 or 10.0 μg/mL. After culturing for 72 h, the minimum lethal dose was selected based on cell survival determined via microscopy.

### BTN1A1-GFP plasmid construction and overexpression

To construct the BTN1A1 overexpression plasmid, full-length open reading frame sequences were amplified from cDNA using PCR and subcloned into the restriction sites of the pEGFP-N1 vector. The following PCR primer pair was used to clone the bovine BTN1A1 open reading frame (Gene ID: NM_174508.2): Forward 5′-CTCGAGATGGCAGTCTTTCCAAACT-3′, encoding a X*ho*I restriction site; Reverse 5′-AAGCTTAGGCACCCCTTGGCTG-3′, encoding a H*ind*III restriction site. Total RNA was extracted from bovine mammary tissue samples using TRIzol reagent (15,596, Invitrogen) and reverse-transcribed to cDNA as a template for PCR. The PCR products were transformed into TOP10 competent cells. Positive clones were selected, and plasmids extracted. After digestion and identification, the BTN1A1-GFP expression vector was sequenced and verified.

The BMEC were counted and seeded at a density of 5 × 10^5^ in 6-well plates and then cultured with lactogenic medium until 70–80% confluence before transient transfection with BTN1A1-GFP expression vector using Lipofectamine 3000 transfection reagent (L3000008, Invitrogen) according to the manufacturer’s protocol. Briefly, the vector DNA and lipofectamine 3000 reagent mixture was prepared by diluting in OptiMEM medium followed by thorough mixing. The mixture was incubated for 15 min at room temperature before addition of the DNA-lipid complex to the cells. After culture for 48 h, washing with PBS, the LD were stained with Nile Red. Abundance of the BTN1A1-GFP fusion protein and LD were determined under an inverted fluorescence microscope to analyze the co-localization of the BTN1A1 and lipid droplet.

### Construction of Cas9/sgRNA expression vectors

Exons 2 and 3 of the bovine *BTN1A1* gene (GenBank ID 282157) were selected as target loci for editing, and sgRNA were designed as reported previously [27] . All potential 20 bp primer sequences followed by 5′-CACC-3′ were scored and analysed based on several factors, including mismatches and the number of off-target sites. Three sgRNA (Table 1) were selected based on their predicted score, synthesised by Invitrogen (Beijing, China), and subcloned into the lentiCRISPR v2 vector (52961, Addgene), resulting in BTN1A1-sgRNA1, BTN1A1-sgRNA2 and BTN1A1-sgRNA3.

**Table 1:** Primers used for BTN knockout and identification

Primer name	Primer sequence (5′→3′)^1^	Primer function
BTN-sgRNA1-F	caccGTTTCCAAACTCCTGCCTCGC	Knockout exon 2 gene of bovine *BTN1A1* gene
BTN-sgRNA1-R	aaacGCGAGGCAGGAGTTTGGAAAC	
BTN-sgRNA2-F	caccGCAGCTGCCCAAGCTGGATTC	Knockout exon 2 gene of bovine *BTN1A1* gene
BTN-sgRNA2-R	aaacGAATCCAGCTTGGGCAGCTGC	
BTN-sgRNA3-F	caccGACCCCCGGAGCCCATCCTGG	Knockout exon 3 gene of bovine *BTN1A1* gene
BTN-sgRNA3-R	aaacCCAGGATGGGCTCCGGGGGTC	
BTN-Test1-F	TGCCTTCTCCTAAGACTCTCTTGG	T7 EI enzyme digestion of exon 2 gene sequence
BTN-Test1-R	GATAATTCGCAGCTCCTTCTCTA	
BTN-Test2-F	TAGAGAAGGAGCTGCGAATTATC	T7 EI enzyme digestion of exon 3 gene sequence
BTN-Test2-R	ATCATCAGAGGCTTTGACCTCCTG	
PPARγ–qPCR-F	CCAAATATCGGTGGGAGTCG	qPCR for *PPAR γ* gene
PPARγ–qPCR-R	ACAGCGAAGGGCTCACTCTC	
ADRP–qPCR-F	CATCTGTTGCAGTTGAACCAC	qPCR for *ADRP* gene
ADRP–qPCR-R	AAGCCGAGGAGACCAGATCA	
FASN–qPCR-F	ACCTCGTGAAGGCTGTGACTCA	qPCR for *FASN* gene
FASN–qPCR-R	TGAGTCGAGGCCAAGGTCTGAA	

^1^cacc and aaac: complementary bases

### Lentivirus generation

The HEK293T/17 cells were seeded in six-well plates at 1 × 10^6^ cells/well. The following day, HEK293T/17 cells were co-transfected with 2 μg of lentiCRISPR v2 -BTN sgRNA 1–3 plasmids, 1.5 μg of psPAX2 plasmid, and 0.5 μg of pMD2.G plasmid. All transfections were carried out with TurboFect reagent (Thermo Scientific) as recommended by the manufacturer. After 8 h (37 °C, 5% CO_2_), the medium was replaced with growth medium supplemented with 10% FBS, and after a further 24 h the medium was changed. Lentiviruses were harvested at 48–72 h, virus-containing media were pooled, centrifuged at 800×*g* for 5 min, and supernatants were used to infect BMEC.

### Cell infection and T7E1 assays

The BMEC were transfected with lentivirus supernatant until 60–70% confluence. After transfecting for 48 h, the medium was replaced with one containing puromycin (5 μg/mL). After puromycin selection of BMEC for 72 h, genomic DNA was isolated using a MiniBEST Universal Genomic DNA Extraction Kit (Takara, China) according to the manufacturer’s instructions and stored − 20 °C until use. PCR amplification of the target gene using primers BTN1A1-Test1 and BTN1A1-Test2 primer (Table 1) was performed using Q5 High-Fidelity DNA Polymerase (MD491S, New England Biolabs) with 35 cycles at 98 °C for 10 s, 65 °C for 20 s, and 72 °C for 20 s.

For T7E1 assays, 200 ng of PCR product was incubated at 95 °C for 5 min in 1× NEBuffer 2, then slowly cooled from 95 °C to 85 °C at a rate of − 2 °C/s, and to 25 °C at a rate of − 0.1 °C/s. After annealing, 5 U of T7 endonuclease I (M0302, New England Biolabs) was added to each sample and reactions were incubated at 37 °C for 15 min. T7E1-digested products were separated on a 2.5% agarose gel, stained with ethidium bromide, and visualised using a gel documentation system (BIO-RAD).

### Generation of individual cell clones and sequencing verification

Individual cell clones were isolated from the positive cell pool by the limiting dilution method as described previously [28]. After transfection with Cas9/sgRNA- BTN1A1–3 expression plasmids as described above, positive cells were diluted to one cell per 100 μL of medium, inoculated into 96-well cell culture plates, and cultured for 10–14 days to obtain single clone colonies. Genomic DNA was isolated from individual clones, and PCR was performed using the BTN1A1-Test primer. PCR products were purified and subjected to sequencing and TA cloning sequencing.

### Real-time quantitative PCR

The WT (normal BMEC) and *BTN1A1*^(−/−)^ BMEC were plated at 50,000 cells per well in 6-well plates for cellular RNA extraction (*n* = 3). After culturing with lactogenic medium for 48 h, total RNA was extracted from BMEC using a TRIzol Plus RNA Purification Kit (12183555, Thermo Fisher Scientific) according to the manufacturer’s protocol. Briefly, first-strand cDNA was synthesised using a Prime Script RT Reagent kit with gDNA Eraser (Takara, Japan), and qPCR analysis was performed using SYBR fast qPCR mix (Takara) and a 7500 fast Real-Time PCR System (ABI). Primers for amplification of target genes by real-time PCR are listed in Table 1. The overall percentage relative mRNA abundance for each gene among all those measured was calculated using the inverse of PCR efficiency raised to ∆Ct. The efficiency of PCR amplification for each gene was calculated using the standard curve method [E 10^(− 1/standard curve slope)^]. The ubiquitously expressed transcript (*UXT*), ribosomal protein S15 (*RPS15*) and ribosomal protein S9 (*RPS9*) were selected as internal control genes for normalization [29].

### Lipid droplet staining

The WT and *BTN1A1*^(−/−)^ BMEC were plated at 30,000 cells per well in 12-well plates for Nile red staining (*n* = 3). After culturing with lactogenic medium for 48 h, LD staining was performed as described previously [30]. Briefly, BMEC were washed twice with phosphate-buffered saline (PBS) for 3 min, fixed with 4% paraformaldehyde for 20 min, and Nile Red (10 μg/mL) added to each well and incubated for 15 min at room temperature. After washing with PBS three times, plates were placed in a Varioskan LUX full-wavelength microplate reader (Thermo Scientific) and fluorescence intensity was measured at an excitation wavelength of 480 nm and an emission wavelength of 575 nm. All experiments were conducted in triplicate.

### Oil red O staining and lipid droplet size determination

The WT and *BTN1A1*^(−/−)^ BMEC were plated at 60,000 cells per 35-mm Glass Bottom Cell Culture Dish (NEST Biotechnology, China) for oil red staining (*n* = 3). After culturing with lactogenic medium for 48 h, Oil-Red-O staining was performed using Oil-Red-O working solution (G1262, Solarbio) with modifications. Briefly, cells were washed thrice with PBS and fixed with 4% paraformaldehyde for 20 min at room temperature. After three washes in PBS, cells were washed with isopropanol and stained with Oil-Red-O working solution (G1260, Solarbio) for 25 min, then washed with PBS, and nuclei re-stained with hematoxylin for 1–2 min. Cells were covered with distilled water and images were visualised using an Olympus IX73 fluorescence microscope equipped with an Olympus DP80 digital camera. Forty LD were randomly selected from each cell culture dish and diameter measured using Cell Sens standard software (version 1.13; Olympus). Average LD diameter values represent the mean size of all 120 LD per group. Lipid droplets were divided into four size groups (0 < size < 2.0 μm, 2.0 μm < size < 2.5 μm, 2.5 μm < size < 3.0 μm and size > 3.0 μm). The number of LD were counted within each of these four sizes.

### Lipid extraction and liquid chromatography-tandem mass spectrometry (LC-MS/MS)

The WT and *BTN1A1*^(−/−)^ BMEC were plated at 150,000 cells per 60-mm in a Cell Culture Dish for lipid extraction (*n* = 6). After culturing with lactogenic medium for 48 h, cells were harvested using trypsin, homogenized in 200 μL of ultra-pure water, mixed with 200 μL of pre-cooled methanol, and 800 μL methyl tertiary-butyl ether. After vortexing and sonication in a low temperature water bath for 20 min, and room temperature for 30 min, samples were centrifuged at 14,000×*g* for 15 min at 4 °C, and the upper organic phase was carefully removed and dried using nitrogen gas. Samples were dissolved in 200 μL of isopropanol/methanol (1/1, v/v) prior to LC-MS/MS detection.

Separation of lipids was performed on an ACQUITY UPLC CSH C18 column (1.7 μm internal diameter, 2.1 mm × 100 mm; Waters Corp.) using an ultra-high-performance liquid chromatography (UHPLC) Nexera LC-30A instrument (Shimadzu Technology, Japan). Samples separated by UHPLC were analysed by a Q-Exactive Plus Mass Spectrometer (Thermo Fisher Scientific) in positive ion mode with a heater temperature of 300 °C, a sheath gas flow rate of 45 arb, an aux gas flow rate of 15 arb, a sweep gas flow rate of 1 arb, a spray voltage of 3.0 kV, a capillary temp of 350 °C, an S-lens RF level of 50%, and an MS1 scan range of 200–1800. Spectra were also obtained in negative ion mode with a heater temperature of 300 °C, a sheath gas flow rate of 45 arb, an aux gas flow rate of 15 arb, a sweep gas flow rate of 1 arb, a spray voltage of 2.5 kV, a capillary temp of 350 °C, an S-lens RF level of 60%, and an MS1 scan range 250–1800.

Peak recognition, lipid identification, peak extraction, peak alignment, and quantitative analysis were performed with Lipid Search software version 4.1 (Thermo Fisher Scientific). Lipid Search software is composed of 8 categories, 300 subclasses, and a database of approximately 1.7 million lipid species. Through the identification algorithm of sub ion, parent ion and neutral loss scanning, the software performs systematic and reliable qualitative analysis of lipids. For data extracted by Lipid Search, lipid molecules with RSD > 30% were deleted. Total peak area was normalised and used for further quantification and analysis. The PLS-DA model scores R^2^ = 0.996 and Q^2^ = 0.975 were obtained by partial least square discriminant analysis of all experimental and QC samples. According to the variable importance in projection (VIP) obtained with the PLS-DA model, influence intensity of each lipid expression pattern was measured, and different lipids with biological significance were obtained. Total phospholipid content was the sum of all peak intensities of phospholipids species identified. Peak intensities of lipid species were summed into their respective classes. The relative percentage of lipid classes was calculated by dividing peak intensities of each classes by total phospholipid peak intensities.

### Statistical analyses

Statistical analysis of all data was performed via SPSS 20.0 (IBM, Armonk, NY, USA). All experiments were conducted in triplicate. Data are reported as Means ± SE. Significant differences were determined using t-tests. All *P* values < 0.05 were considered statistically significant. The most differentially expressed phospholipid species in the lipidomics data were identified at a cutoff value *P* < 0.05, VIP > 1, Fold change> 2.5 or < 0.5.

## Results

The β-casein gene was highly-expressed in cultured BMEC (Additional file 1: Fig. S1). Most cells survived at the dose of 2.5 μg/mL puromycin. When dose of puromycin was greater than 5.0 μg/mL, most cells died. Thus, the minimum lethal dose of puromycin against BMEC was 5.0 μg/mL (Additional file 1: Fig. S2). This concentration was used for subsequent BMEC selection.

### BTN1A1 protein and lipid droplet co-localization

**Fig. 1:**
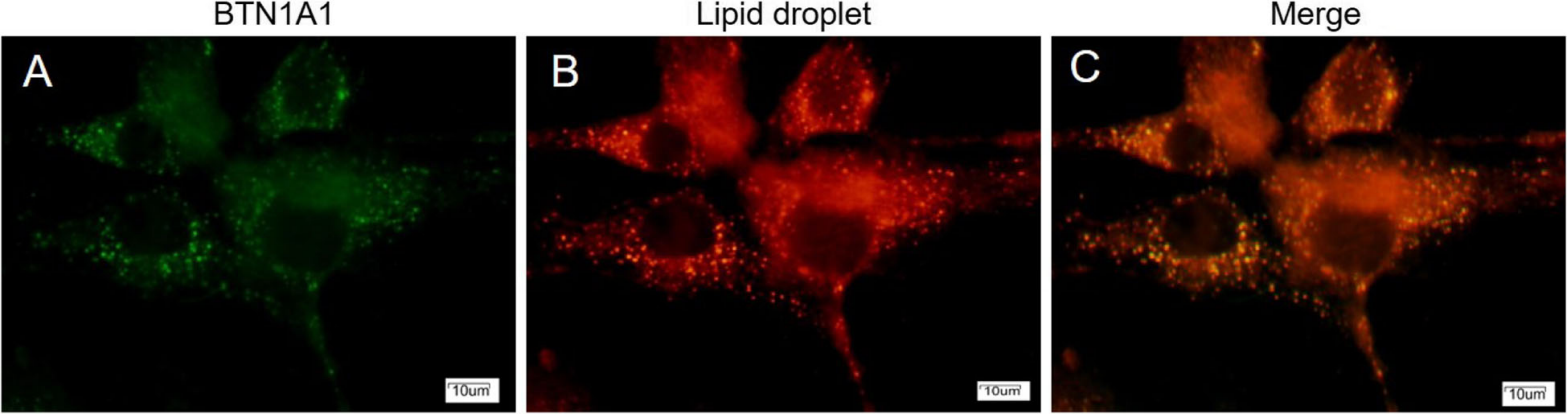
Co-localization of the BTN1A1 and lipid droplets in bovine mammary epithelial cells (BMEC). Abundance of BTN1A1-GFP fusion protein and lipid droplets was observed under an inverted fluorescence microscope. **a** BTN1A1-GFP fusion protein. **b** Lipid droplets staining with nile red. **c** BTN1A1-GFP and lipid droplet co-localization

The BTN1A1-GFP fusion protein led to green fluorescence in the BMEC (Fig. 1a). The LD stained red fluorescence (Fig. 1b). Both BTN1A1 and LD were co-localized in the cytoplasm (Fig. 1c).

### CRISPR/Cas9-mediated knockout of the *BTN1A1* gene

**Fig. 2:**
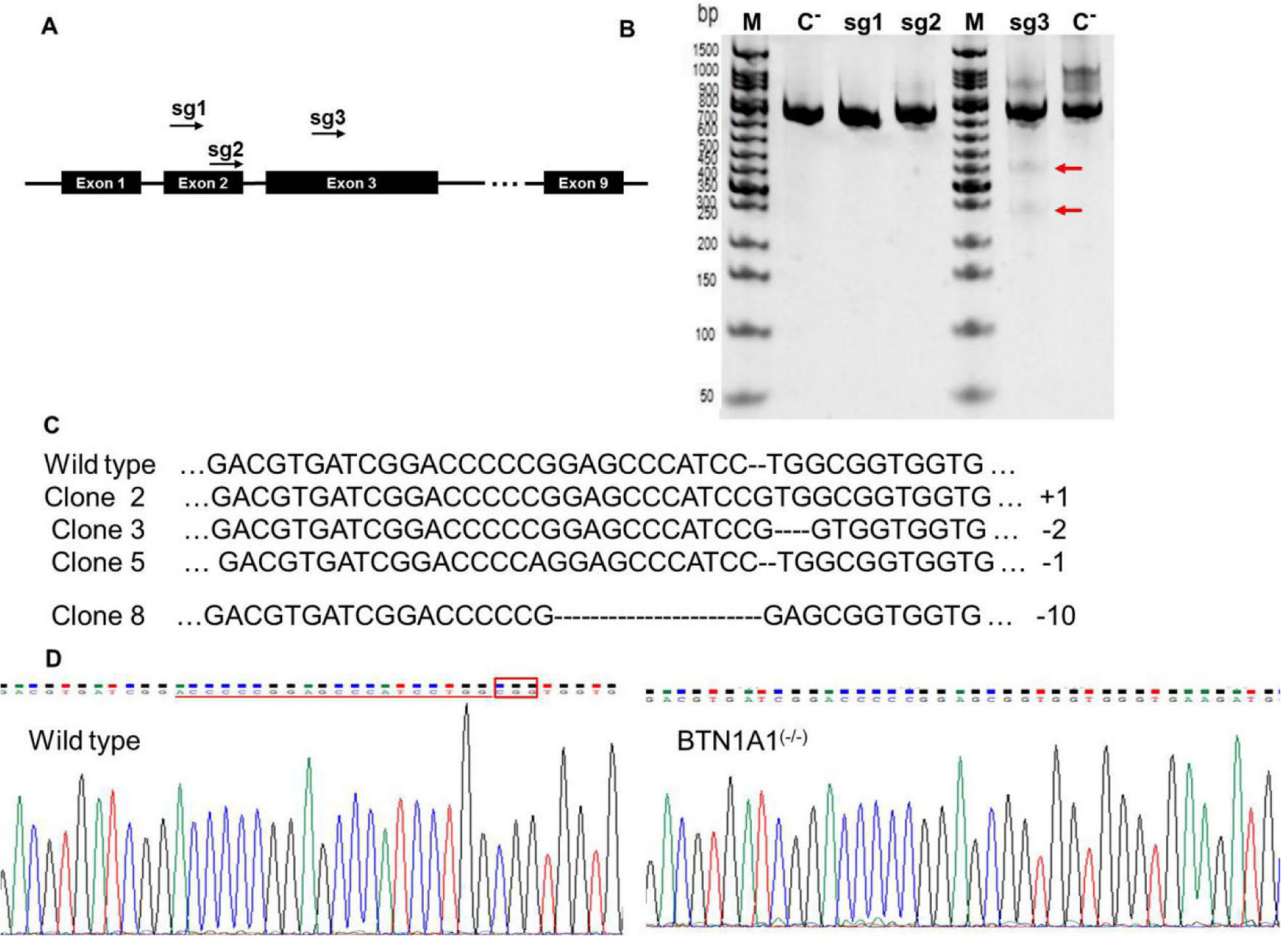
Selection and identification of a *BTN1A1* knockout clone. **a** Design of three sgRNA (sgRNA1, sgRNA2 and sgRNA3) targeting exons 2 and 3 of the *BTN1A1* gene. **b** After puromycin selection of transfected cells, T7EI assays were performed to evaluate the genome editing effect. Lane M, 50 bp DNA markers; C−, PCR products of wild-type (WT) cells used as a negative control. **c** Sequences of modified *BTN1A1* alleles in mutant clones. **d** TA clone sequencing showing a10-bp deletion the *BTN1A1* gene in clone 8. The single horizontal red line indicates the deleted base pair next to the protospacer adjacent motif

We designed three different sgRNA targeting exons 2 and 3 of the *BTN1A1* gene (Fig. 2a). T7E1 cleavage assays yielded two DNA bands for sgRNA3 (Fig. 2b), indicating that sgRNA3 successfully edited the genomic DNA of selected cells. Based on the T7E1 cleavage results, *BTN1A1*-sgRNA3 was used in subsequent experiments and infected into BMEC to generate single clones. We obtained 4 single clones following gene editing. To assess knockout efficiency, the BTN1A1 protein levels in different mutants were measured by western blotting (Additional file 1: Fig. S3). Clone 8 was deemed to have sufficient BTN1A1 knockout, and sequencing revealed this clone included a 10-bp deletion in the target sequence in both alleles (Fig. 2c and d). Clone 8 was, thus, used as the homozygous *BTN1A1*^(−/−)^ clone in further experiments.

### *BTN1A1* knockout affects abundance of lipogenic genes

**Fig. 3:**
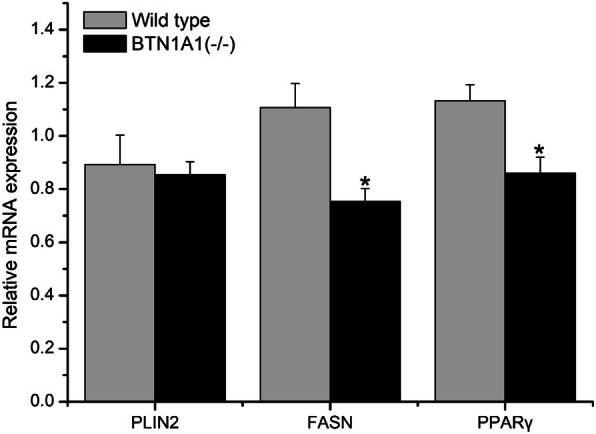
Effect of knockout of *BTN1A1* on lipogenic mRNA abundance in bovine mammary epithelial cells (BMEC) (*n* = 3). Data are reported as Means ± SE. Total RNA was isolated using TRIzol reagent and the relative abundance of target genes was measured using real-time PCR.WT, wild-type; *BTN1A1*^(−/−)^, *BTN1A1* gene knockout cell (*P < 0.05)

Knockout of *BTN1A1*downregulated (*P* < 0.05) mRNA abundance of *FASN* and *PPARG* (Fig. 3), but had no significant effect (*P* > 0.05) on *PLIN2* abundance.

### *BTN1A1* knockout decreases lipid droplet content and diameter

**Fig. 4:**
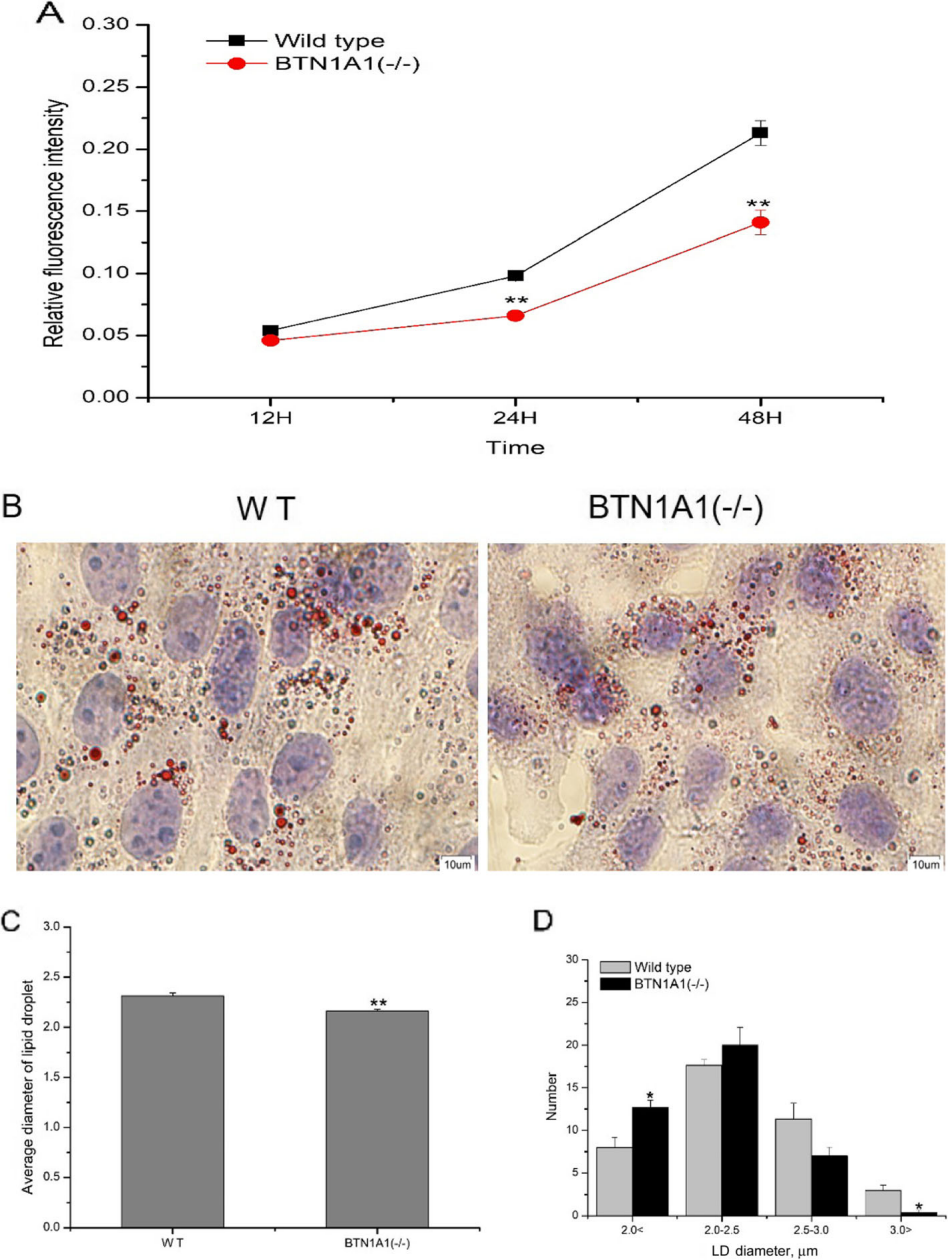
Effect of knockout of *BTN1A1* on lipid droplet (LD) formation in bovine mammary epithelial cells (BMEC). All experiments were conducted in triplicate. All LD visualised within cells were measured using CellSens Entry software (version 1.7; Olympus). Data are reported as Means ± SE. **a** Nile Red staining of LD was performed after 12, 24 and 48 h of cell culture. Relative fluorescence intensity of LD was quantified at an excitation wavelength of 480 nm and an emission wavelength of 575 nm in a full-wavelength microplate reader. **b** LD were stained red by Oil-Red-O, and nuclei were re-stained blue by hematoxylin. **c** The average LD diameter (μm) was determined by analysis 120 randomly chosen LD per group. **d** Number of LD of different sizes. Lipid droplets were divided into four size groups (0 < size < 2.0 μm, 2.0 μm < size < 2.5 μm, 2.5 μm < size < 3.0 μm and size > 3.0 μm). Number of LD was counted within the four sizes. WT, wild-type; *BTN1A1*^(−/−)^, *BTN1A1* gene knockout cells (*P < 0.05,**P < 0.01)

After *BTN1A1* knockout, Nile Red staining revealed that compared with the WT group, LD formation was decreased. With increasing culture time, LD content of *BTN1A1*^(−/−)^ cells decreased from 1065 (12 h) to 644 (48 h), demonstrating a time-dependent effect (Fig. 4a). After knockout of *BTN1A1*, average LD diameter decreased from 2.31 μm in WT cells to 2.16 μm in *BTN1A1*^(−/−)^ cells (*P* < 0.01; Fig. 4b Fig. 4c). Number of LD in a specific diameter range was determined for WT and *BTN1A1*^(−/−)^ groups (Fig.4d). Compared with WT, the number of small LD (diameter < 2.0 μm) was greater, and large LD (diameter > 3.0 μm) lower in *BTN1A1*^(−/−)^ cells (*P* < 0.05).

### Knockout of *BTN1A1* affects the cell membrane phospholipid composition

**Fig. 5:**
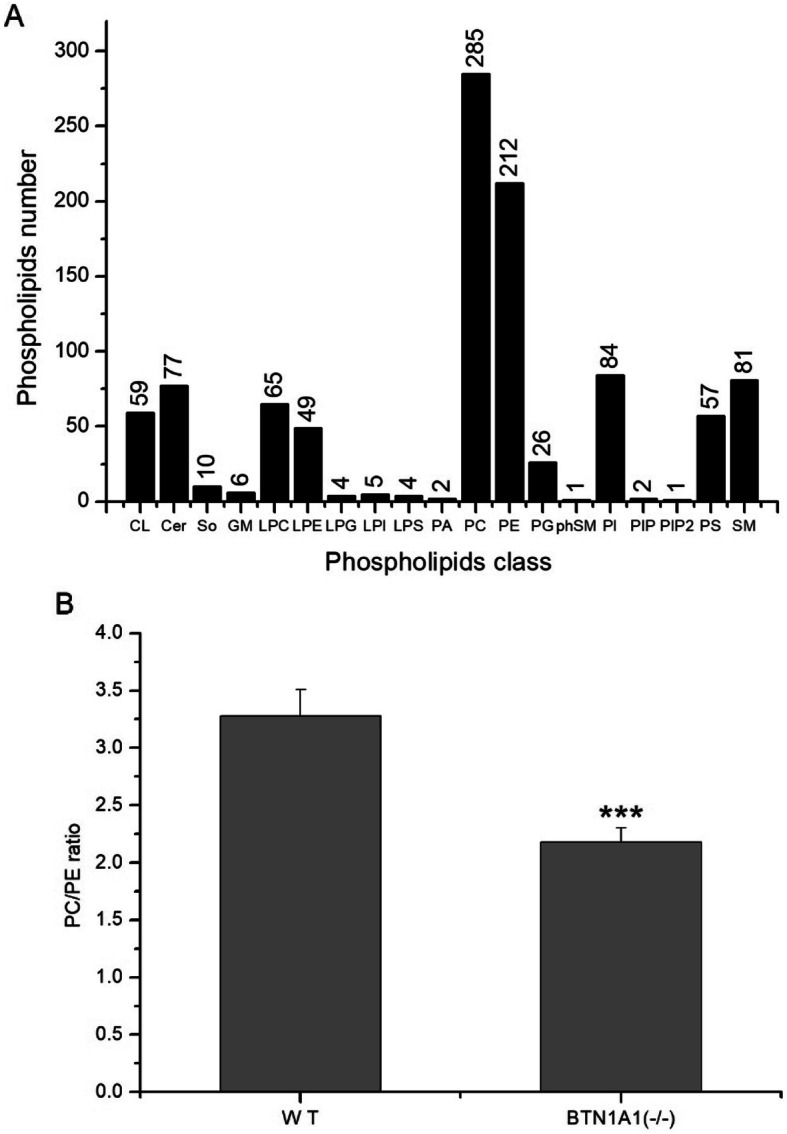
Effect of knockout of *BTN1A1* on phospholipid numbers and relative composition in bovine mammary epithelial cells (BMEC). Lipids of WT and *BTN1A1*^(−/−)^ cells (*n* = 6) were extracted and analysed by LC-MS/MS. Lipid identification and quantitative analysis were performed with lipid Search software version 4.1. Data are reported as Means ± SE. **a** Identified phospholipid numbers in bovine mammary epithelial cells (BMEC). **b** Ratio of PC/PE, calculated as relative percentage of phosphatidylcholine vs. phosphatidylethanolamine in *BTN1A1*^(−/−)^ vs. WT cells. (***P < 0.001)

A total of 1030 phospholipid species were identified in WT and *BTN1A1*^(−/−)^ cells by LCMS/MS analysis (Fig. 5a, Additional file 1 Table S1). PC and PE were the most predominant lipids classes (285 and 212, respectively). Peak intensities of lipid species were summed into their respective classes. The percentage of the polar lipid class out of total phospholipids in both WT and *BTN1A1*^(−/−)^ cell is shown in Table 2. The PC, PE, PI, PS and SM accounted for more than 95% of total phospholipids. Compared with WT cells, there was greater relative percentage of PE (22.55% vs. 16.90%) and lower of PC (49.24% vs. 55.35%) in the *BTN1A1*^(−/−)^ cell membrane (*P* < 0.01, Table 2) resulting in a significantly lower ratio of PC/PE (*P* < 0.001, Fig. 5b). The most different phospholipid species are shown in Table 3, with LPE(18:1) increasing to 4.13-fold and PC(44:1) decreasing to 0.12-fold.

**Table 2:** Relative percentage of phospholipids classes between WT and *BTN1A1*^(−/−)^ bovine mammary epithelial cells. Value are presented as LS means ± SEM (*n* = 6)

	PC	Relative % of phospholipids^1^			SM
		PE	PI	PS	
W T	55.35 ± 1.47^a^	16.90 ± 0.93^a^	3.30 ± 0.09^a^	5.42 ± 0.32^a^	15.59 ± 1.23^a^
*BTN1A1*^(−/−)^	49.24 ± 1.28^b^	22.55 ± 0.90^b^	3.30 ± 0.23^a^	5.27 ± 0.27^a^	15.71 ± 1.03^a^

^ab^Within the same column differ (*P* < 0.05)

^1^*PC* phosphatidylcholine, *PE* phosphatidylethanolamine, *PI* phosphatidylinositol, *PS* phosphatidylserine, *SM* sphingomyelin

**Table 3:** Most-significantly affected phospholipid species in bovine mammary epithelial cells containing *BTN1A1*^(−/−)^

Species^1^	Fold-change^2^	*sn*-1^3^	*sn*-2	*sn*-3	*sn*-4
LPE(18:1)	4.13	(18:1)			
Cer(d38:1)	3.19	(d18:1)	(20:0)		
PE(40:3)	3.12	(18:1)	(22:2)		
Cer(d36:1)	2.87	(d18:1)	(18:0)		
PE(38:3p)	2.77	(18:0p)	(20:3)		
Cer(d34:0)	2.55	(d18:0)	(16:0)		
CL(74:2)	0.47	(18:1)	(18:0)	(18:0)	(20:1)
PC(40:1)	0.47	(18:1)	(22:0)		
CL(66:5)	0.46	(16:1)	(16:1)	(16:1)	(18:2)
CL(68:6)	0.44	(18:2)	(16:1)	(16:1)	(18:2)
SM(d43:3)	0.41	(d43:3)			
PC(40:1e)	0.39	(20:0e)	(20:1)		
PC(42:2)	0.33	(18:1)	(24:1)		
PE(42:1)	0.32	(20:1)	(22:0)		
PE(44:2)	0.30	(26:1)	(18:1)		
PC(42:1)	0.21	(18:1)	(24:0)		
PC(40:0)	0.15	(16:0)	(24:0)		
PC(44:2)	0.14	(26:1)	(18:1)		
PC(44:1)	0.12	(26:0)	(18:1)		

^1^The most differentially expressed phospholipid species were identified with cutoff values of VIP > 1, Fold change> 2.5 or < 0.5 in *BTN1A1*^(−/−)^ mammary cells relative to wild-type

^2^Ratio of phospholipid peak intensity in *BTN1A1*^(−/−)^ mammary cells relative to wild-type

^3^*sn* denotes fatty acid side chains of phospholipids

## Discussion

Members of the butyrophilin (BTN) gene family are attracting increased attention because they may play multifunctional roles in a variety of physiological processes including lactation, regulation of T cells in the immune system, and autoimmune diseases [31]. The BTN1A1 protein is associated with the secretion of milk fat. It is highly-expressed in bovine [17] and caprine [32] mammary gland during lactation. In the present study, the high mRNA abundance of β-casein confirmed that BMEC maintained lactogenic capacity expected of mammary cells. Subcellular co-localization analyses revealed that BTN1A1 was bound to cytoplasmic LD [18]. Knockout of *BTN1A1* in BMEC demonstrated not only an important role of this protein in determining LD size, but also a role in cell phospholipid composition.

The CRISPR/Cas9 system has advantages compared with other genome editing technologies such as transcription activator-like effector nuclease and zinc finger nuclease approaches, including ease of use, high efficiency, and adaptability to different organisms [33, 34]. In the present study, use of the lentiCRISPR v2 vector, which is constructed around a 3rd generation lentiviral backbone, allowed simultaneous infection/transfection of Cas9 and sgRNA followed by selection via puromycin resistance [35]. Editing via CRISP/Cas9 can leave a variety of mutations because when the target region is amplified, and PCR products are denatured and renatured, there are mismatches at the target site. As shown in Fig. 2b, the presence of two shorter bands in agarose gel electrophoresis following product cleavage suggests that CRISPR/Cas9-sgRNA3 successfully introduced insertion or deletion mutations in the genomic target DNA. After selection of a single clone, DNA sequencing confirmed a 10 bp deletion mutation in the *BTN1A1* exon (Fig. 2c and d). Furthermore, BTN1A protein abundance was completely blocked in the *BTN1A1*^(−/−)^ clone. These results indicate that the CRISPR/Cas9 system successfully knocked out the *BTN1A1* gene, yielding a homozygous clone.

In the present study, knockout of *BTN1A1* gene in BMEC decreased content and diameter of LD. In liver [36, 37] and adipose [38] cells, the two main factors controlling LD size are phospholipids and proteins on their surface. BTN1A1 is a type I membrane protein that is incorporated into the surface membrane coat surrounding LD [18]. Thus, the fact that *BTN1A1* deficiency decreased the size of LD in the present study underscores its importance in the context of LD fusion.

The nuclear receptor *PPARG* is a member of the PPAR family, which mediates lipid accumulation through the induction of lipogenic genes such as *PLIN2* and *FASN* in mammary cells [26, 39, 40]. Thus, the lower abundance of *PPARG* and *FASN* suggests a regulatory role for these genes in LD formation. Some reports also link PPAR activation via phospholipids. For instance, sphingomyelin treatment regulated the expression of *PPARG* [41, 42]. Shi et al. [43] reported that activation of PPARD affected abundance of genes related to fatty acid metabolism (e.g. *FASN*, *PLIN2*) in goat MEC. In the present study, knockout of BTN1A1 affected *PPARG* abundance and phospholipid classes, which we speculate provides some evidence of a role for phospholipids on PPAR activity in BMEC. Clearly, additional experiments are needed to better define the mechanisms linking phospholipids and PPAR in the overall process of LD formation.

It is well-known that cellular membranes are made primarily with phospholipids, and alterations in their availability may enable better dispersion of triacylglycerol in the form of smaller MFG. In turn, such effect may greatly affect the size of MFG [10]. It has been reported that the size of LD in BMEC is determined by the phospholipid composition of the cell membrane [9, 44]. The ratio of PC/PE has been suggested as a predictor for LD size. We evaluated the effect of BTN1A1 knockout on membrane phospholipid composition and found that the percentage of PC decreased and the PE increased (Table 2), resulting in a lower PC/PE ratio (Fig. 5b) in the *BTN1A1*^(−/−)^ cells. Consistent with these results, SCD-deficient *C. elegans* displayed decreased LD size with lower percentage of PC, higher PE, and lower PC/PE ratio [45].

Phosphatidylethanolamine can be transformed into PC by the action of transferases, and PE could be produced by decarboxylation of PS [46]. Thus, factors affecting phospholipid synthesis may be involved in the regulation of LD size. The chain length, unsaturation, and polarity of phospholipids affect the diffusion and transport rate of phospholipids in the membrane [10]. We also found that the most different phospholipid species contained monounsaturated fatty acids in the sn-1 position primarily (C16:1 or C18:1, Table 3). These results suggest that a variety of phospholipids may play roles in regulating the size of LD.

## Conclusions

The abundance of *BTN1A1* gene in ruminant mammary cells is critical for formation of lipid droplets, i.e. regulate the content and size of LD in BMEC. Besides its role in lipid droplet formation, BTN1A1 exerts some degree of control on transcription of lipogenic genes and the profile of cell membrane phospholipids. Although the present study cannot explain the exact mechanisms of BTN1A1 action, the determination of LD size within ruminant mammary cells seems determined by complex regulatory mechanisms including milk fat-globule membrane protein abundance and phospholipid composition of the membranes.

## Supplementary information

**Additional file 1: Figure S1.** mRNA abundance of β-casein gene in BMEC. **Figure S2.** Optimum lethal dose of puromycin against BMEC. **Figure S3.** BTN1A1 protein expression levels in different mutants. **Table S1.** Phospholipid lipidomics data by LC-MS/MS

## Data Availability

The data during and /or analysed during the current study are available from the corresponding author on reasonable request.

## Abbreviations

BTN1A1: Butyrophilin subfamily 1 member A1
LD: Lipid droplets
MEC: Mammary epithelial cells
MFG: Milk fat globule
CRISPR/Cas9: CRISPR and CRISPR-associated 9 (Cas9)
PLIN2: Adipophilin
FASN: Fatty acid synthase
PPARG: Peroxisome proliferator-activated receptor-gamma
PS: Phosphatidylserine
PI: Phosphatidylinositol
PE: Phosphatidylethanolamine
PC: Phosphatidylcholine
SM: Sphingomyelin
LPE: Lysophosphatidylethanolamine
Cer: Ceramides
CL: Cardiolipin
