# Supplementary material for: Knockout of butyrophilin subfamily 1 member A1 (BTN1A1) alters lipid droplet formation and phospholipid composition in bovine mammary epithelial cells
Source: J Anim Sci Biotechnol. 2020 Jul 3;11:72. doi: 10.1186/s40104-020-00479-6 (PMC7333294; doi:10.1186/s40104-020-00479-6)
Supplement: Supplementary file 1 — Additional file 1: Figure S1. mRNA abundance of β-casein gene in BMEC. Figure S2. Optimum lethal dose of puromycin against BMEC. Figure S3. BTN1A1 protein expression levels in different mutants. Table S1. Phospholipid lipidomics data by LC-MS/MS [file 40104_2020_479_MOESM1_ESM.doc]

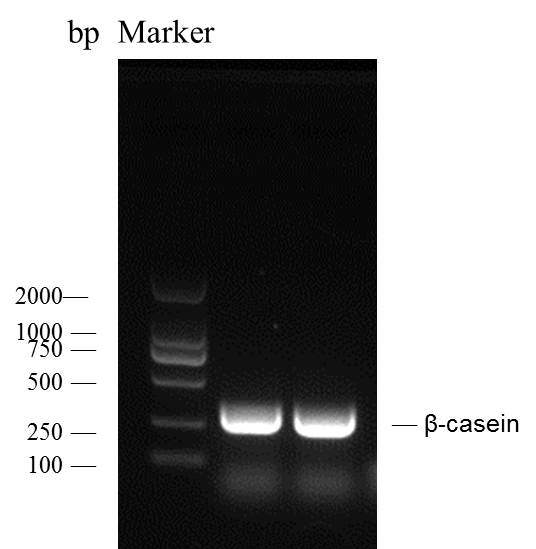


**Fig. S1** The mRNA expression of *β-casein* gene in BMECs. BMECs were cultured in lactogenic medium and β-casein mRNA expression was measured by RT-PCR. The PCR primer pair: Forward 5′- GAGATTGTGGAAAGCCTTTC-3’,Reverse 5′- CTTTCAGTAAAGGGCTCAAC-3’.


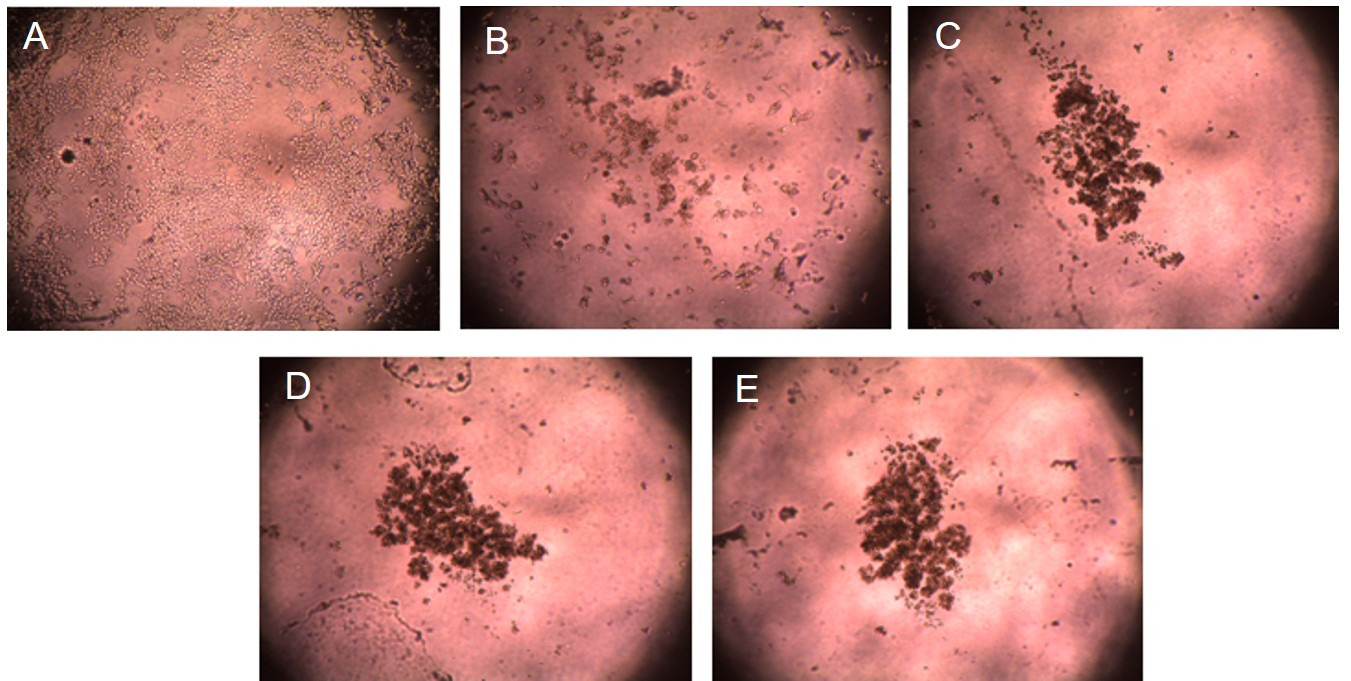


**Fig. S2** The optimum lethal dose of puromycin against BMECs. (A) 0 μ g/mL puromycin；(B) 2.5 μ g/mL puromycin；(C) 5.0 μ g/mL puromycin；(D) 7.5 μ g/mL puromycin；(E) 10 μ g/mL puromycin


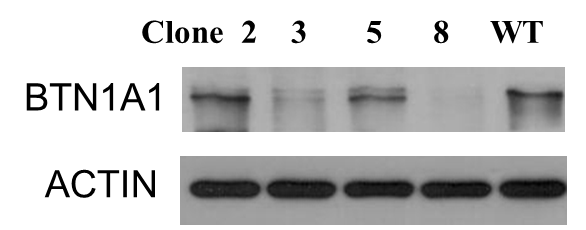


**Fig. S3** BTN1A1 protein expression levels in different BMEC mutants. Proteins were extracted from transfected cells using RIPA lysis and extraction buffer (89900; Thermo). Protein concentration was measured using a Pierce BCA assay kit (23225; Thermo). A 50 μg sample of total protein per lane was separated by sodium dodecyl sulphate-polyacrylamide gel electrophoresis (SDS-PAGE) and transferred to a polyvinylidene fluoride (PVDF) membrane. After blocking with 5% skim milk for 1 h, membranes were incubated with primary antibody at 4°C overnight. The primary antibodies used in this study were anti-β-actin (1:5000, ym3028, Immunoway) and anti-BTN1A1 (1:50, BM5509, Acris). After washing five times with TBST, membranes were probed with horseradish peroxidase (HRP)-conjugated AffiniPure Goat Anti-Mouse IgG (H+L); 1:2000, Proteintech) for 1 h at room temperature.Immunoblots was performed using the standard ECL procedure（Amersham™ ECL™ Prime,GE,USA）.


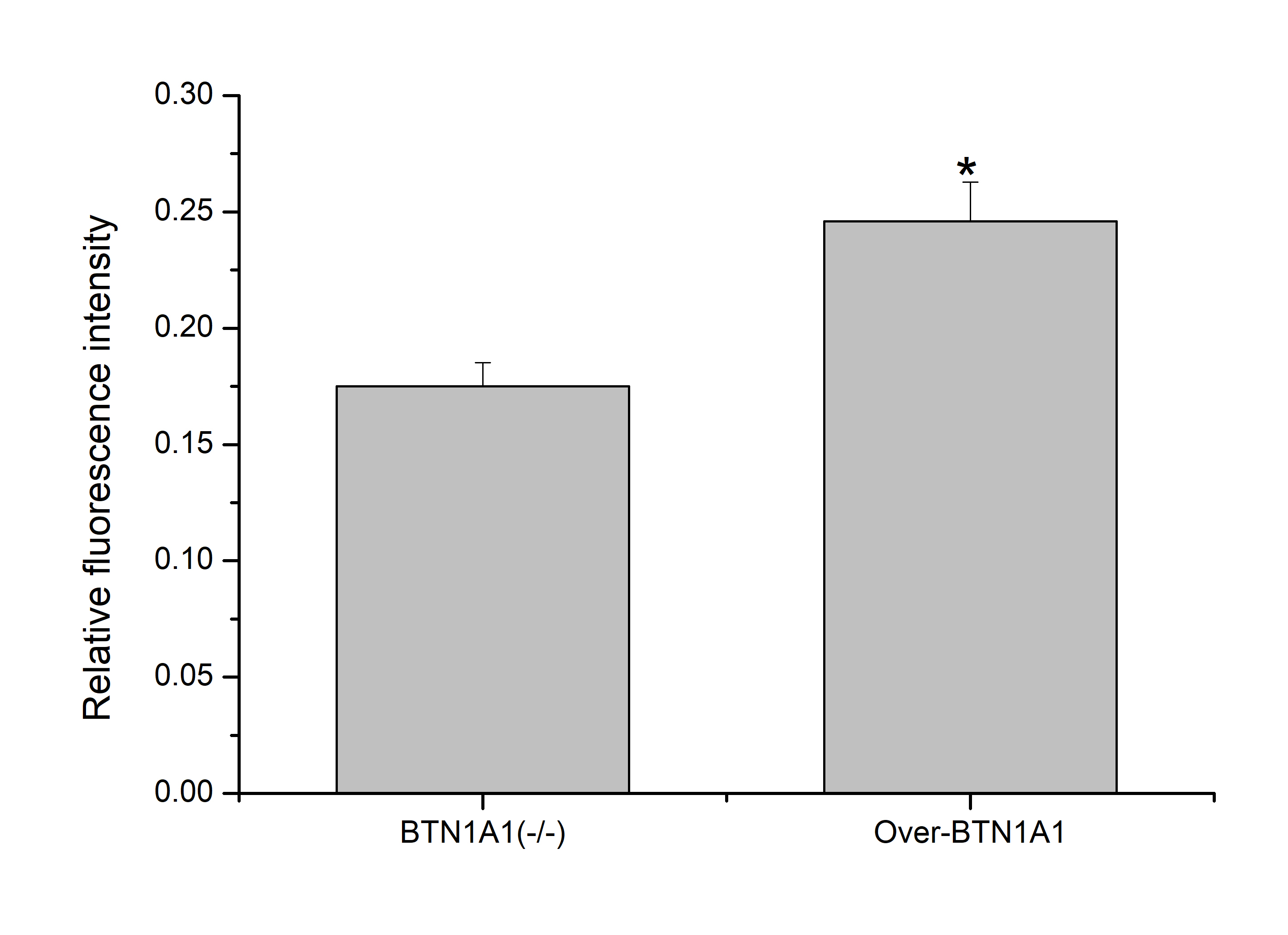


**Fig. S4** Over-expression *BTN1A1* gene in BTN1A1 knockout cells. The *BTN1A1*^(-/-)^ cells were seeded at a density of 5×10^5^ in 6-well plates and transiently transfection with pEGFP-N1 (BTN1A1(-/-) group) and BTN1A1-GFP expression vector (Over-BTN1A1 group) using Lipofectamine 3000 transfection reagent. After cultured 48h, LD staining was performed, and the fluorescence intensity was measured at an excitation wavelength of 480 nm and an emission wavelength of 575 nm using a Varioskan LUX full-wavelength microplate reader (Thermo Scientific).
